# A synthetic segmentation dataset generator using a 3D modeling framework and raycaster: a mining industry application

**DOI:** 10.3389/frai.2024.1453931

**Published:** 2024-12-13

**Authors:** Wilhelm Johannes Kilian, Jaco Prinsloo, Jan Vosloo, Stéphan Taljaard

**Affiliations:** Faculty of Engineering, North-West University, Potchefstroom, South Africa

**Keywords:** deep learning, computer vision, image segmentation, deep-level mining, real applications in engineering

## Abstract

Many industries utilize deep learning methods to increase efficiency and reduce costs. One of these methods, image segmentation, is used for object detection and recognition in localization and mapping. Segmentation models are trained using labeled datasets; however, manually creating datasets for every application, including deep-level mining, is time-consuming and typically expensive. Recently, many papers have shown that using synthetic datasets (digital recreations of real-world scenes) for training produces highly-accurate segmentation models. This paper proposes a synthetic segmentation dataset generator using a 3D modeling framework and raycaster. The generator was applied to a deep-level mining case study and produced a dataset containing labeled images of scenes typically found in this environment, therefore removing the requirement to create the dataset manually. Validation showed high accuracy segmentation after model training using the generated dataset (compared to other applications that use real-world datasets). Furthermore, the generator can be customized to produce datasets for many other applications.

## 1 Introduction

Many economies are in various stages of Industry 4.0 adoption. The Industry 4.0 paradigm can be described as the digitization of complex industrial systems through the implementation and integration of advanced technologies (Prinsloo et al., 2019). This enables initiatives such as predictive maintenance, remote real-time monitoring and control, and process optimization, which are the value drivers for the adoption of Industry 4.0 (Stark et al., 2019).

The South African deep-level mining industry is in the process of shifting toward Industry 4.0 to utilize these value drivers (Prinsloo et al., 2019; Stark et al., 2019; Dhamija, 2022). Digital twins (DTs), one of the technologies enabling these value drivers, are digital representations of physical systems with cyber-physical connections (Aheleroff et al., 2021).

DTs enable access to sensor measurement data and system metadata within the system's three-dimensional (3D) context (Aheleroff et al., 2021). However, DTs are time-consuming to create manually and existing semi-automatic generation methods, e.g., light detection and ranging (LiDAR), which are typically expensive and require specialized equipment (Baek et al., 2022).

Localization and mapping is the process of capturing information about the surrounding area such that a digital map is created (Wei and Akinci, 2019). This technique has been used to generate DTs instead of creating them manually. However, simply using a 360° camera to capture all the tunnels in a deep-level mine is not feasible, as even a short 10-minute recording using this type of camera can result in large data storage requirements (Bidgoli et al., 2020).

In recent years, deep learning methods have shown promising results in the shift toward the Industry 4.0 paradigm (Radanliev et al., 2022; Ahmad et al., 2022; Khayyam et al., 2020). One of these deep learning methods, namely image segmentation, has been used for localization and mapping problems (Manettas et al., 2021). Image segmentation is a method used to divide images into smaller regions to locate and identify certain objects of interest (Minaee et al., 2022). Utilizing image segmentation, the required data storage size can be reduced for localization and mapping. Segmentation can identify whether an image contains valuable information, determining whether an image needs to be stored.

Deep learning image segmentation models require labeled datasets to be trained. The higher the quality of the dataset, the better the chance of high-accuracy segmentation. Dataset quality is typically determined by factors such as size, label accuracy, and variance (to avoid overfitting) (Duan et al., 2022). However, these labeled datasets are almost always created manually (Lee et al., 2022).

Manual creation of these labeled datasets is an extremely time-consuming process and, typically, expensive as many people are often employed to parallelize its creation (Lee et al., 2022). At the time of this research, no labeled dataset for deep-level mining applications could be found, and manually creating it would be inefficient in terms of time and cost. Recently, the creation and use of synthetic datasets (digital recreations of real-world scenes which are automatically labeled) for the training of deep learning models have shown promising results (Manettas et al., 2021; Greff et al., 2022).

Much research has been invested into image segmentation, as can be seen in the paper by Minaee et al., where they surveyed over 100 recent papers (Minaee et al., 2022). Most of these papers focused on applications containing bicycles, people, vehicles, fire hydrants, and other objects found in typical street scenes. Many of these papers applied the technology for object identification and localization in self-driving vehicles, which require the identification of common objects, for which many datasets already exist. It is more challenging to find datasets for uncommon scenarios, and in some cases, they do not even exist, as was the case for deep-level mines at the time of this study.

Minaee et al. (2022) also summarized the most well-known metrics used for image segmentation models, in order of proven resulting performance, such as Pixel Accuracy (PA), Intersection over Union (IoU), and the Dice coefficient (also known as the F1 score).

Pixel Accuracy (PA) is simply a measurement of the proportion of correctly- to incorrectly-labeled pixels. This rather simple metric, unfortunately, suffers from the class imbalance problem. Intersection over Union (IoU) considers the overlapped segmentation area versus the total area of the predicted and ground truth labels. It circumvents the class imbalance issue and provides better entity distinction in instance segmentation, making it widely used in various papers (Wang et al., 2023; He et al., 2021). The Dice coefficient, or F1 score, assesses twice the overlap of predicted and ground truth labels against their total area (Minaee et al., 2022). Valued for its balanced measure and insensitivity to global differences, it does not skew based on object class frequencies or object sizes (Wu et al., 2021; Milletari et al., 2016).

Cheng et al. (2020) considered another metric, Panoptic Quality (PQ), for their segmentation model named Panoptic-Deeplab. They used the Cityscapes (Cordts et al., 2016), Mapillary Vistas (Neuhold et al., 2017), and COCO (Lin et al., 2014) datasets for training and validation. All of these are real-world datasets of objects typically found in traffic images and other common scenarios.

**Figure 1:**
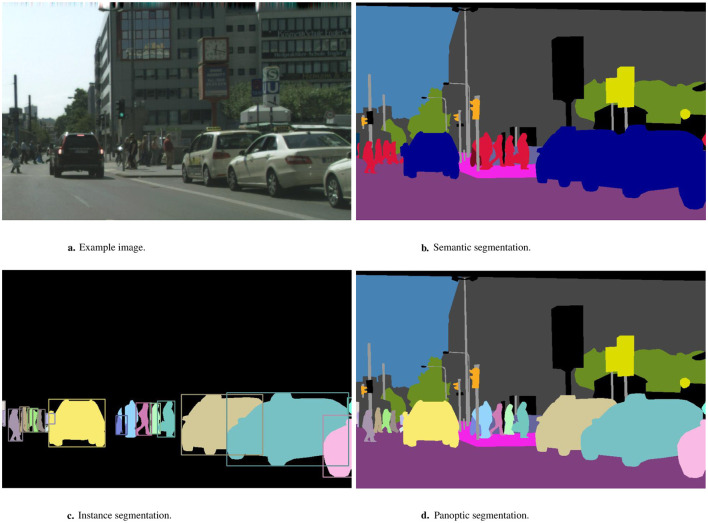
Segmentation techniques (Kirillov et al., 2019). **(a)** Example image. **(b)** Semantic segmentation. **(c)** Instance segmentation. **(d)** Panoptic segmentation.

PQ, originally proposed by Kirillov et al. (2019), is the average IoU over each class, divided by the sum of the true positive and false positive matches. The class imbalance problem is avoided by averaging the IoU and giving each class equal weight, while incorrect labels are penalized by dividing by the false matches. Kirillov et al. proposed this metric to combine semantic and instance segmentation approaches to create panoptic segmentation, as shown in Figure 1. Semantic segmentation (Figure 1b) groups all pixels in an image together in classes represented by one color per class, instance segmentation (Figure 1c) identifies unique instances of objects and assigns each a different color, and panoptic segmentation (Figure 1d) is a combination of the two.

Kirillov et al. (2023) introduced the Segment Anything Model (SAM) in a more recent study. Their study aimed to create a model capable of segmenting any image. Their model has been shown to segment a wide variety of images. However, object classes not present in their dataset are only labeled as generic objects. Therefore, it is not suitable for segmenting and labeling objects of interest when they are uncommon and not present in the training dataset they used.

Zanella et al. (2021) proposed a synthetic dataset generator for the semantic segmentation of cable-like objects. They utilized domain randomization to create datasets that are generalisable across different environments. Domain randomization consists of creating images of the objects of interest with many different backgrounds. However, this generalisability has been shown to reduce segmentation performance for specific problems not well represented in the generated dataset (Choi et al., 2021). They utilized the Dice coefficient for the evaluation of their proposed generator.

Kong et al. (2020) produced a synthetic dataset for urban environments. However, their study focused on high-resolution satellite imagery for building footprint segmentation. They combined their synthetic labeled dataset with the limited real-world datasets available to train their models. This combination resulted in high-accuracy segmentation performance. Kong et al. used IoU to evaluate the performance of their segmentation model, which is a sensitive evaluation metric compared to PQ (Kirillov et al., 2019).

Greff et al. (2022) recently introduced a dataset generator using Blender (a 3D computer graphics tool), which can be used to generate synthetic scenes of many different types of real-world scenarios. Their framework, however, includes features such as depth mapping, optical flow, segmentation, and surface normals. This causes the generator to require significant computational power to generate the synthetic dataset as it computes many other properties of the synthetic scenes.

Many papers have discussed the reality gap when using synthetic data to train deep learning models (Wang et al., 2022). The reality gap is the real-world effects that are not represented in the synthetic data because it would be so complex to model that creating the dataset becomes unfeasible. Some examples of these real-world effects are object deformation, foggy weather, and complex lighting conditions (Wu et al., 2021).

Synthetic datasets also tend to suffer from overfitting, which occurs when the dataset does not have enough variance to produce a segmentation model that performs well on unseen real-world data (Hosna et al., 2022). Recently, authors have utilized transfer learning during the training phases to overcome this reality gap.

Transfer learning is the process of training a deep learning model using real-world datasets to gain knowledge about the real domain and then fine-tuning the model to the specific application by training it in a second phase using the synthetic dataset (Manettas et al., 2021). Transfer learning has been shown to produce highly-accurate segmentation models (Wu et al., 2021; Wang et al., 2022).

In their paper, Manettas et al. (2021) utilized transfer learning in computer-vision tasks in manufacturing. They initialized their model's weights using a model pre-trained on the ImageNet dataset. The ImageNet dataset, at the time of their study, contained 1.2 million real-world labeled images. After initialization, they trained the model on their relatively small synthetic dataset (300 labeled images per class) and achieved segmentation performance comparable to models trained using a real-world dataset created specifically for the application and even outperformed some.

Wu et al. (2021) improved commodity segmentation using transfer learning. They used the weights of the ResNet model to initialize their model and then used a dataset they created manually, consisting of 2,800 labeled commodity images, to fine-tune for their application. A significant improvement was found when they compared the segmentation accuracy of the model, which was trained using transfer learning, to one trained without transfer learning. Wu et al. (2021) utilized IoU and the Dice coefficient to analyze performance.

For this paper, instance segmentation will be used, as only the predefined classes of interest are regarded as regions of interest. The IoU and PQ metrics will be used during the validation of the dataset generator proposed in the following section. The IoU metric was chosen as it circumvents the class imbalance problem prevalent in metrics such as PA (Wang et al., 2023). The Dice coefficient was not chosen, as it is very similar to the IoU metric. The PQ metric was chosen as it improves upon the IoU metric by considering the true positive and false positive matches (Kirillov et al., 2019). However, both will still be used for comparison purposes during the validation process.

Digital twins, a technology enabling the value drivers of Industry 4.0, are time-consuming to create manually, and existing semi-automatic generation methods are typically expensive and require specialized equipment (Baek et al., 2022). Localization and mapping applications can be used to generate DTs. One of the methods used in localization and mapping is image segmentation. However, the manual creation of the datasets used for the training of image segmentation deep learning models is time-consuming and typically expensive (Lee et al., 2022).

Existing papers have explored the use of synthetic datasets for image segmentation. However, none of them focused on the cost-efficient automatic generation of instance segmentation datasets (Greff et al., 2022; Manettas et al., 2021; Zanella et al., 2021). Therefore, there is a need for a method to automatically generate synthetic, labeled datasets in a cost-efficient manner for many different applications. This paper proposes a synthetic dataset generator using a 3D-modeling framework and raycaster. A South African deep-level mining case study is then used to verify the proposed generator.

## 2 Synthetic segmentation dataset generator

This section is split into three parts. First, the synthetic scene creation process will be proposed. Then, the automatic labeling process using a raycaster will be proposed. Finally, the strategy that will be used to validate the generator will be discussed.

### 2.1 Synthetic scene generation

#### 2.1.1 Elements in a scene

A real-world scene and the elements (objects and phenomena) within it must be analyzed before the scene can be synthetically recreated. It is unfeasible in most applications to precisely model every aspect of a real-world scene due to the reality gap (Wang et al., 2022). Therefore, elements of interest must be chosen to synthesize the real-world scene accurately enough for the relevant application (Greff et al., 2022).

The elements of interest and the required accuracy with which real-world elements must be modeled depend on the application and constraints. The object classes which should be recognized in the trained segmentation model are the highest priority. Other fundamental parts of the real-world scene, such as the walls and floors in a corridor when considering an indoor environment, should also be included as elements of interest.

The object classes (the first category of elements previously mentioned) must be defined before creating the segmentation dataset. Some examples of object classes are ladders, vehicles, windows, and doors. More detailed object classes may be chosen, such as different vehicle types or door shapes. The level of detail for the chosen classes must be based on the specific application.

The number of chosen object classes should be restricted. Segmentation performance has been shown to degrade when the number of object classes to identify passes a certain threshold (Wang et al., 2022). This threshold is determined by the available computational resources and training time available.

Phenomena (the second category of elements) should also be analyzed, such as object interaction and ambient lighting in real-world scenes. This can be done by posing questions such as (1) is the scene well-lit, (2) are there objects moving throughout the scene, and (3) is it expected that solid objects cross each other's boundaries?

An answer to the last question could be that solid objects solely occupy their own space, but there are situations where, for example, a pipe has been placed such that it passes through a wall from one room to another. Another phenomenon to consider is the perspective of one moving through the real-world scenes, as the recreation of it should replicate the real scenario as accurately as possible.

These are only a few examples of phenomena that should be analyzed as they introduce some of the nuances of the real-world scene into the synthetic scene, therefore increasing the possibility of high-performance segmentation. Phenomena that do not significantly increase the similarity of the synthetic scene to the real-world scene should be ignored. The generation of the synthetic scenes can begin once the analysis of the real-world scenes has been completed.

#### 2.1.2 3D modeling framework

Many open-source 3D models exist for various object classes (pipes, compressors, doors, stairways, etc.). These 3D models can be utilized to digitally represent small parts of real-world scenes, which removes the requirement to create them manually and may increase modeling accuracy (if the open-source model is a more realistic representation of the real-world object in comparison to a 3D model that can be created manually in a reasonable amount of time).

These open-source models are typically used in 3D modeling frameworks and computer graphics engines to create virtual scenes, such as the visual aspect of DTs and augmented reality experiences (Lv et al., 2022). These 3D models are stored in many different data formats, such as the stereolithography (STL) and Wavefront OBJ formats. The modeling framework selection method where these 3D models will be utilized is discussed next.

A 3D modeling framework should be chosen based on the combination of outcomes from the analysis of each relevant category described in Table 1. A category is considered irrelevant when it does not significantly affect the application.

**Table 1:** Categories for analyzing 3D modeling frameworks for an application.

**Category**	**Description**
3D model imports and exports	The import and export of 3D models in various formats to increase generation productivity.
Raycasting	The outline of visible objects in each perspective should be identifiable to create the labeled dataset.
Usability and flexibility	Does the framework have comprehensive documentation, an active community, and can generate various scenes?
Advanced physics and simulation	Does the framework support object interactions that aim to mimic real-world phenomena, such as water flow?
Scalability and hardware requirements	How complex can the scene become while balancing the hardware requirements to be feasible for the application?
Licensing and cost	Is the framework free to use?

**Figure 2:**
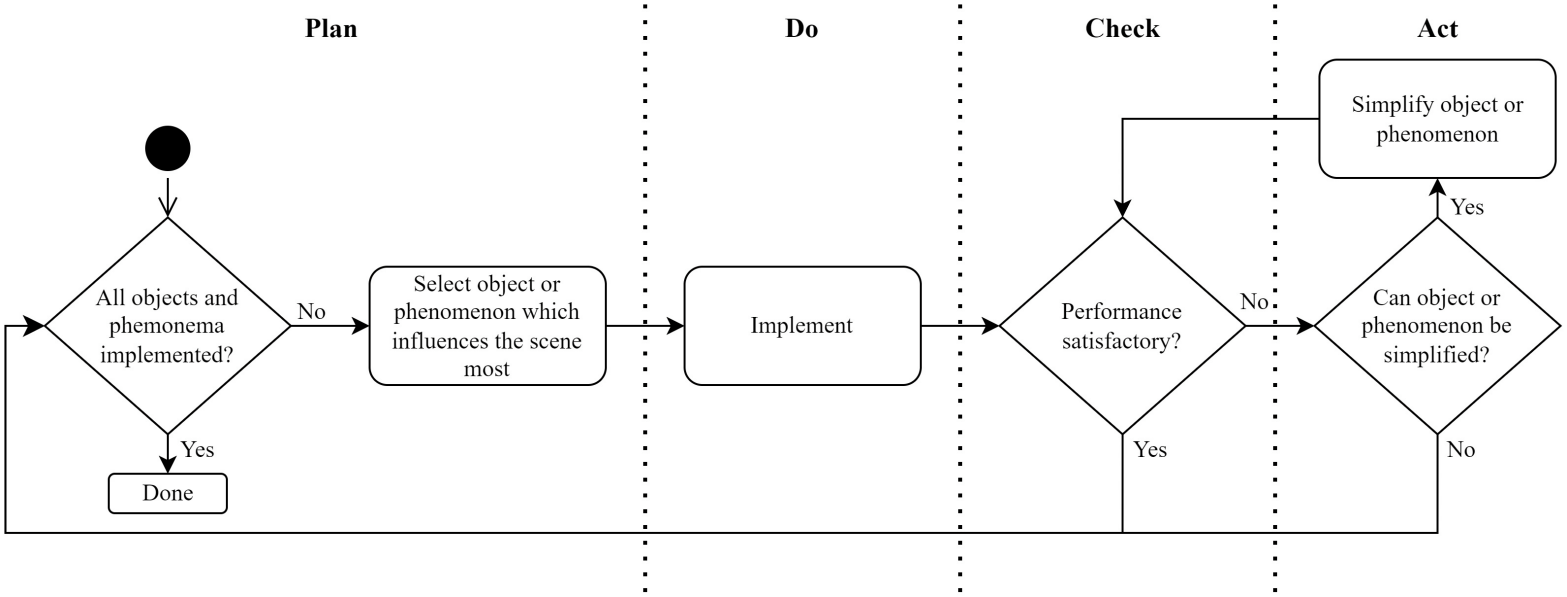
Synthetic scene implementation method.

Once the modeling framework has been chosen, the synthetic scenes can be designed and generated. Creating the synthetic scenes comprises implementing the phenomena and placing the objects previously identified in real-world scenes. The full implementation method has been abstracted into the flow diagram shown in Figure 2 to be relevant for many applications and modeling frameworks.

The implementation method is based on the Plan, Do, Check, Act (PDCA) cycle, which forms part of the ISO 9001 standard (Dentch, 2016). The first step is to select the unimplemented object or phenomenon that would change the scene most significantly. After it has been selected, the object or phenomenon is implemented in the previously chosen 3D modeling framework.

Implementing the object consists of importing the object's geometry into the virtual scene and applying a texture to represent the object's surface. The object's geometry is imported from the open-source file obtained in the chosen format, such as STL or OBJ. The choice of the texture to show on the surface of the object depends on what type of object is being loaded. However, many open-source textures (typically stored as 2D images) are available.

The loaded object is then rendered into the scene, and its scale is updated to ensure a realistic representation of the real-world scene. Following this, the performance of the resulting scene is then analyzed to determine whether the newly implemented object or phenomenon should be simplified. Once the performance is satisfactory, the next most significant object or phenomenon should be implemented.

This process continues until the synthetic scene is populated with as many objects and phenomena present in the real-world scene as possible, such that the performance enables feasible dataset creation times. The objects in the synthetic scenes should not be placed exactly as seen in the real world; otherwise, the training would be prone to overfitting. Placement should rather be pseudo-random (within certain constraints) to increase the dataset variance and different perspectives of the objects.

After all feasible information has been added to the synthetic scene, a randomization process is used to create new unique scenes within the rules defined during the analysis phase. The automatic labeling process of these pseudo-random variations of the synthetically created scene is proposed in the following section.

### 2.2 Automatic labeling using a raycaster

All 3D modeling frameworks have a method of visualizing the scenes generated (Greff et al., 2022). This visualization is typically implemented using a camera object with a predefined location within and perspective of the scene. Now, consider a person walking down a street, their perspective can be mimicked by placing a camera in a synthetic scene at the height of an average person. Altering the camera's location would then mimic the person walking through the scene while altering the camera's pitch, roll, and yaw would mimic head rotation.

This imitation of the change of perspective will be used to generate multiple different images from one scene variation. Consequently, multiple scene variations will be produced, increasing the potential size of the generated dataset. Deep learning datasets are required to contain many different variations of inputs and expected outputs to the problem that needs to be solved. A dataset that is too small may result in overfitting, while a dataset that is too large may result in over-generalization during training.

**Figure 3:**
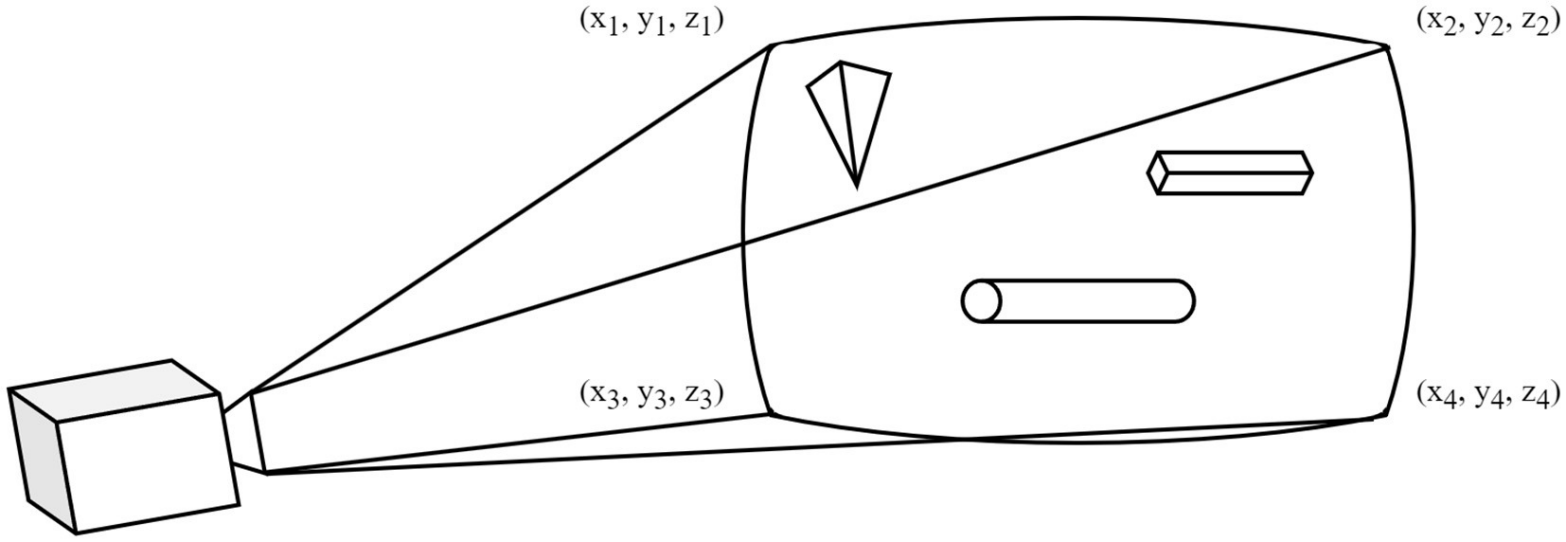
Camera perspective example.

The automatic labeling will be applied to each perspective sampled in the synthetic scenes. This process will determine the outlines (polygons) of all the objects visible within the current perspective. The perspective of a camera object is shown with lines indicating the extremes of the perspective in Figure 3. Objects (a pyramid, cylinder, and cuboid) have also been added to the scene for illustration purposes.

The automatic labeling process starts by defining the limits of the camera perspective (each corner represented by an (*x*_*i*_, *y*_*i*_, *z*_*i*_) value in Figure 3) and then capturing the current camera's perspective as a 2D image. Therefore, the generated 3D scene is converted to a 2D image of the current perspective of the scene.

**Figure 4:**
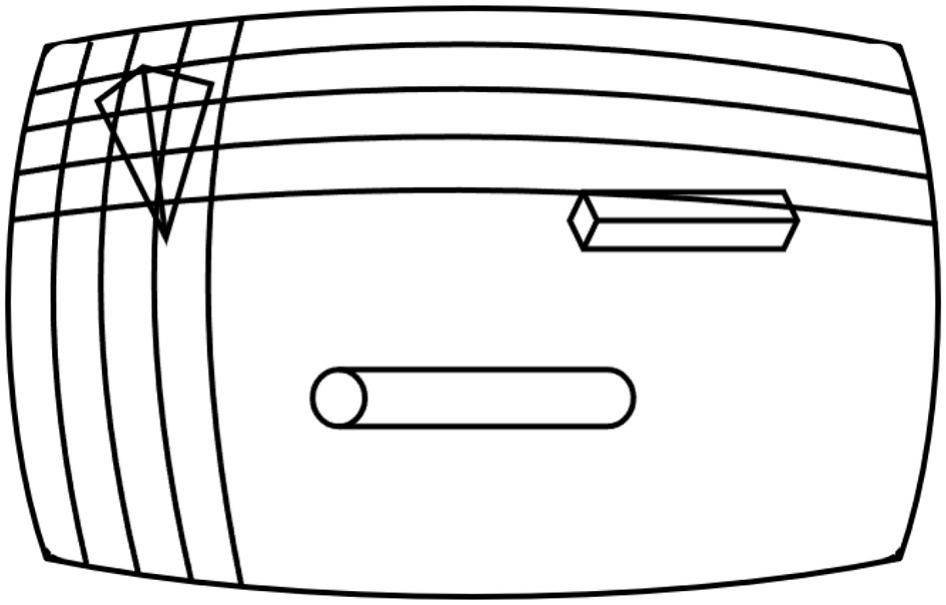
Raycasting matrix from the camera's perspective.

After the 2D image has been captured, the current camera perspective is divided into an *M* × *N* matrix for raycasting. Raycasting is the process of tracing a straight line sent from the origin of the camera's perspective toward a chosen coordinate to find the first object in its path. Figure 4 represents a simplified example of the raycasting matrix for visualization.

Each element in this matrix, represented by a vector with its origin at the camera's location, is raycast iteratively to identify what, if any, object is present. This *M* × *N* matrix is then converted to sets of polygons (segmentation labels for irregularly shaped objects, i.e., not squares or circles) using Algorithm 1.

**Algorithm 1:** *M* × *N* raycast matrix result to polygon labels.

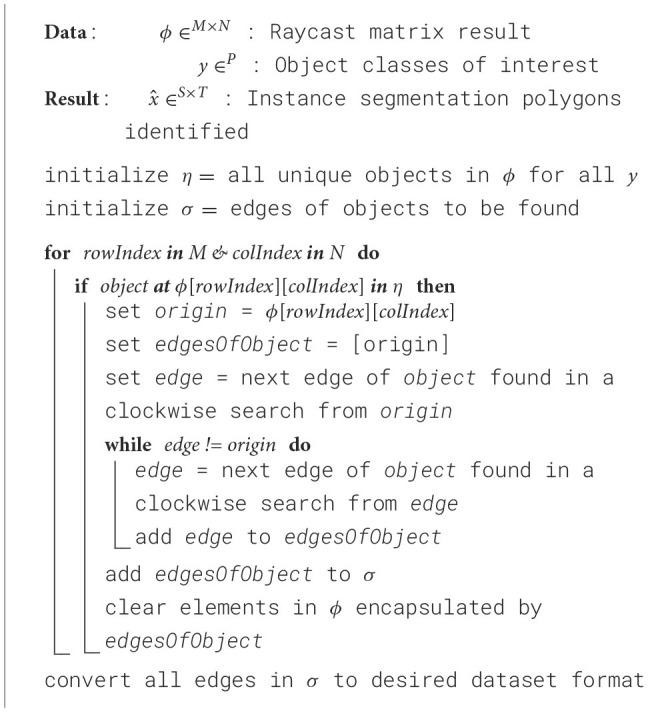

The raycast result matrix is iterated from top-left to bottom-right to identify all the individual instances of each predefined object class. When an element in the matrix matches an object class, the elements around it are tested in a clockwise approach (starting from the element directly to the right of it) to determine if their raycast result matches the same object class and ID (to differentiate between instances of object classes).

This process continues until the full outline of the current object is completed. Once all matrix elements are identified as part of an outline or do not contain the desired object classes, we convert these outlines into sets of (*x, y*) coordinates.

These coordinate sets and the 2D perspective image are then added to the dataset. The labeling format should integrate well with the selected 3D framework and model training software, as described in Section 2.1.2. Repeating this process for all perspectives and scene variations will yield a synthetic instance segmentation dataset for the desired application. Increasing the scene variations and perspectives will expand the final dataset.

The proposed method of synthetic scene generation and automatic labeling comprises the overarching methodology for creating synthetic segmentation datasets designed to be broadly applicable. The validation strategy is discussed in the following section.

### 2.3 Validation strategy: image segmentation using transfer learning

A methodology has been proposed for synthetic dataset generation. However, its effectiveness in segmentation performance must be evaluated using a validation strategy. This strategy involves applying a transfer learning approach to train a segmentation model on the generated dataset and then using evaluation metrics to assess performance. Transfer learning leverages pre-trained models, freezing inner layers to retain learned features while modifying the final layer's weights using the new dataset. It circumvents the time-consuming and typically costly process of training a model from scratch.

The segmentation performance of the trained models will be analyzed using the evaluation metrics discussed in the introduction, namely, Intersection over Union (IoU) and Panoptic Quality (PQ). IoU is calculated as the area of overlap of the ground truth labels (A) and the predicted labels (B) divided by the area of the union of the ground truth labels and the predicted labels for each object instance, as shown in Equation 1.


(1) $$\begin{array}{l} IoU=\frac{\left|A\cap B\right|}{\left|A\cup B\right|} \end{array}$$

PQ, shown to be a more stable metric than IoU (Mao et al., 2023), is calculated as the sum of the IoU for each object instance divided by the sum of the true positive (TP), false positive (FP), and false negative (FN) matches as shown in Equation 2.


(2) $$PQ=\frac{\sum IoU}{\left|TP\right|+\frac{1}{2}\left|FP\right|+\frac{1}{2}\left|FN\right|}$$

This validation strategy will be used to determine whether applying the proposed methodology creates datasets that, when used in a transfer learning approach, show increasing IoU and PQ performance metrics over each validation epoch. The results of applying the methodology and validation strategy are discussed in the following section.

## 3 Results and discussion

A methodology was proposed in the previous section for a synthetic segmentation dataset generator using a 3D modeling framework and raycaster. This section will present and discuss each major result of the methodology's application to a deep-level mining case study. The results from the methodology's validation will be presented at the end of this section.

### 3.1 Synthetic scenes

**Figure 5:**
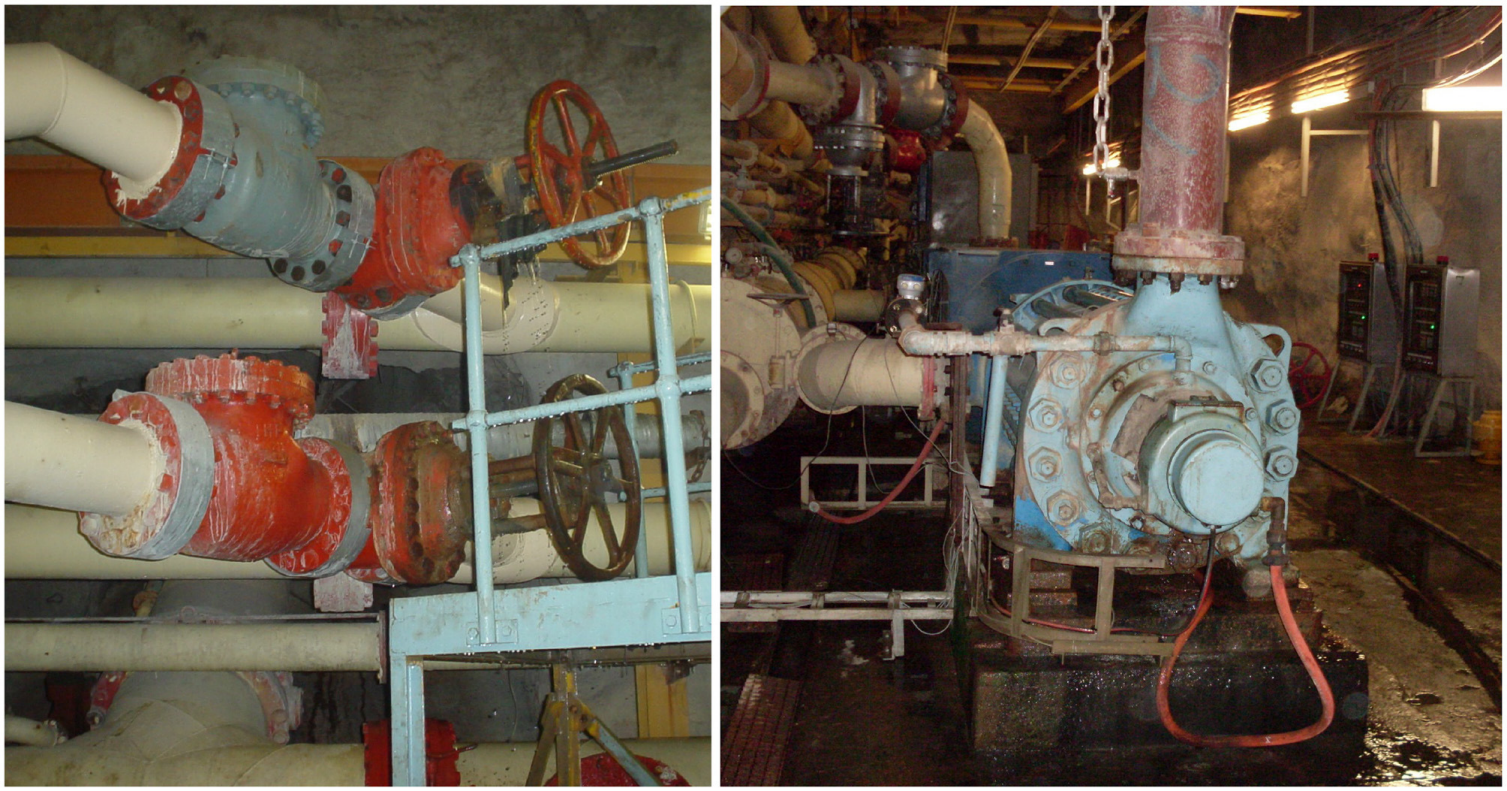
Real-world images of the case study deep-level mine.

The first part of the proposed synthetic scene creation is the analysis of the case study's real-world scenes. Figure 5 shows two images captured in the case study deep-level mine during the analysis phase. The analysis of these and other sample images showed that the objects typically found are pipes, valves, and various pumps and compressors. It was also found that inconsistent lighting conditions are present.

The 3D modeling framework chosen to be used in the generation of the synthetic scenes was Three.js (referred to hereafter as “the framework”). The framework was chosen due to its characteristics in each category described in Table 1, with the results listed below.

- 3D model imports and exports: the framework supports many different import formats.
- Raycasting: the framework includes a camera and raycasting class to efficiently perform the required changing of perspectives and raycast analysis.
- Usability and flexibility: comprehensive documentation and an active community ensure high usability. The framework is an abstraction of the WebGL API (used to render interactive 3D graphics in most web browsers), which decreases the implementation time of the synthetic scene generation while still ensuring flexible customization.
- Advanced physics and simulation: these are supported by the framework but are not relevant to the current application and, therefore, are not considered.
- Scalability and hardware requirements: as the framework is an abstraction of the WebGL API, the scalability and hardware are mostly affected by implementation efficacy. The choice in this category is not affected by costly computations of advanced physics and simulations as they are not required.
- Licensing and cost: The open-source framework aligns with the low-cost requirement of the application.

Popular alternatives to the framework are Unity and Blender. These include highly-advanced physics and simulation features. However, these features (amongst others) are not required by the current application and would only increase the computational requirements. Unity also requires the purchase of costly licenses.

**Figure 6:**
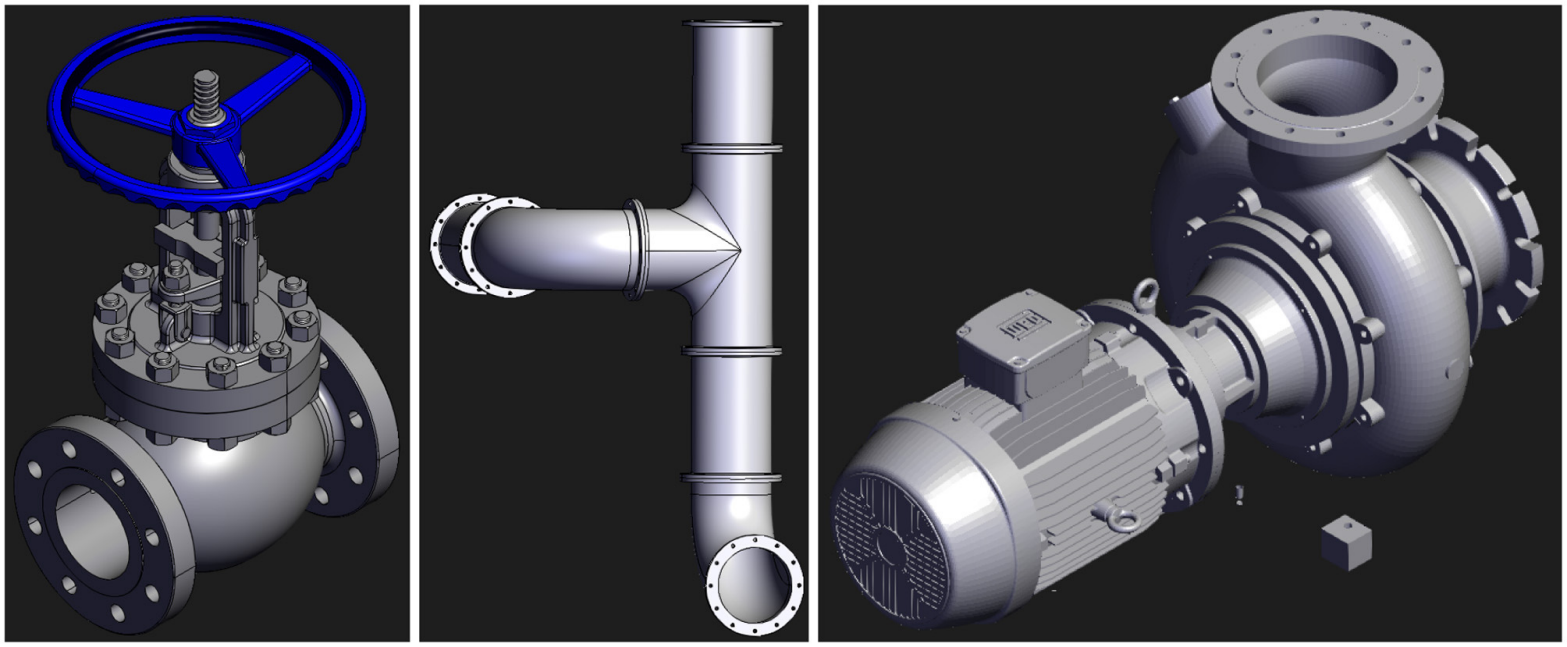
Open-source 3D models for objects of interest.

The next step in the method is to find 3D models that can represent the real-world objects in the scene. As discussed in the analysis of the scenes in Section 2.1, some of the objects of interest include pipes, valves, and centrifugal pumps. Open-source 3D models for each of these object types are shown in Figure 6.

The final stage of scene generation uses the flow diagram in Figure 2, the chosen 3D modeling framework, and open-source 3D models. During the “Act” stage, an oversimplification that could compromise valuable training information should be avoided. The fidelity of a 3D model, typically defined by the count of triangular faces representing its surface, impacts the complexity of the raycast computation. Therefore, it is important to find a balance between model fidelity and raycast performance.

**Figure 7:**
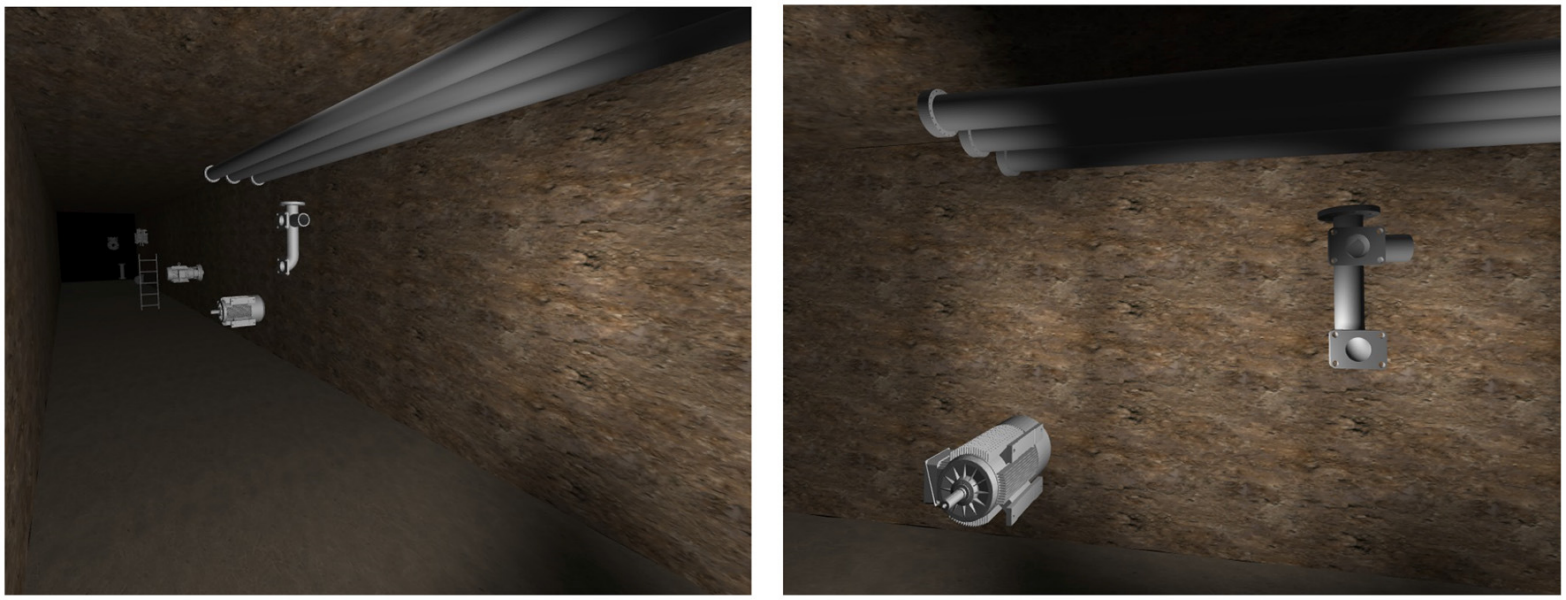
Two different perspectives of a generated synthetic scene.

The images in Figure 7 show the result of applying the flow diagram using the chosen 3D modeling framework and open-source 3D models. The first image in Figure 7 shows the tunnel length generated (with textured walls) and the 3D models placed at pseudo-random positions. The second image shows a different perspective of the same generated synthetic scene.

Certain informed assumptions were required to increase the likelihood that the generated synthetic dataset produces performant segmentation results by avoiding unnecessarily high levels of variance (Greff et al., 2022). Datasets are typically created for a specific application, as this ensures that they contain the required information for efficient segmentation model training (Minaee et al., 2022). Although this limits the generalizability of the specific dataset created, the proposed generation method is generalizable for many applications. This can be seen in the first steps of the proposed method, where the specific application's real-world scenes are analyzed. Therefore, the chosen scene can be varied as required.

### 3.2 Automatic labeling

**Figure 8:**
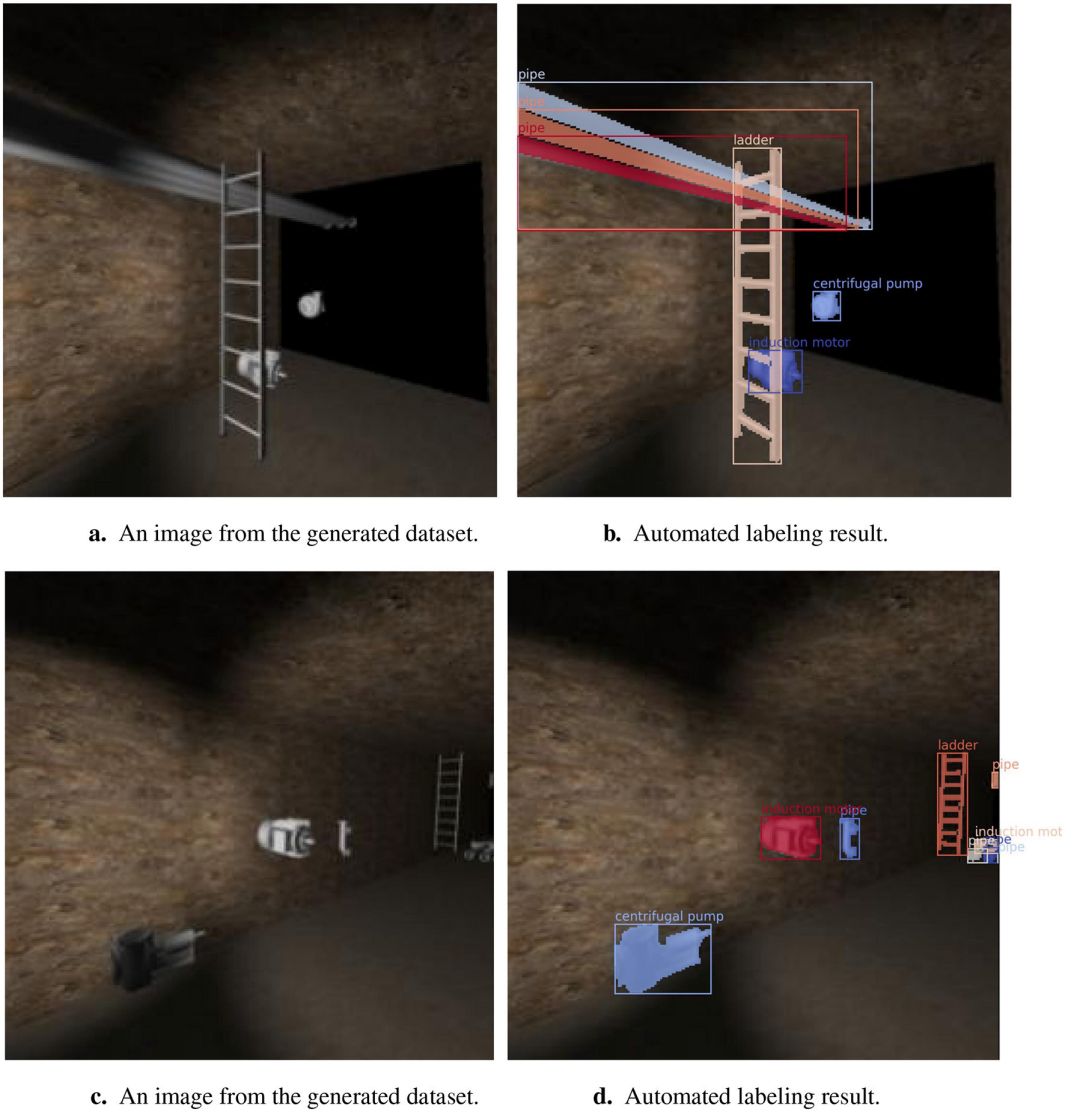
Automated labeling results for images in the generated dataset. **(a)** An image from the generated dataset. **(b)** Automated labeling result. **(c)** An image from the generated dataset. **(d)** Automated labeling result.

This process was implemented using the framework's camera and raycast functionality. Once each perspective was generated, it was divided into a 256 × 256 matrix as shown in Figure 4, and each element was raycast to obtain the object present. Once all the elements in the matrix were raycast, the polygon edges were determined using Algorithm 1. Figures 8a, c show images from the generated dataset and Figures 8b, d the labeled images from the automated raycasting process.

Three pipes, a ladder, an induction motor, and a centrifugal pump have been automatically segmented and labeled in Figure 8b. A centrifugal pump, induction motors, pipes, and a ladder were automatically segmented in Figure 8d. This and other similar generated and labeled perspectives will be used in the transfer learning process to train the deep learning segmentation model in the following section.

### 3.3 Validation results

The dataset created contains six object classes: valves, pipes, ladders, induction motors, heat exchangers, and centrifugal pumps. The dataset consists of 329 automatically-labeled images. Two of the labeled images in the dataset are shown in Figure 8.

**Figure 9:**
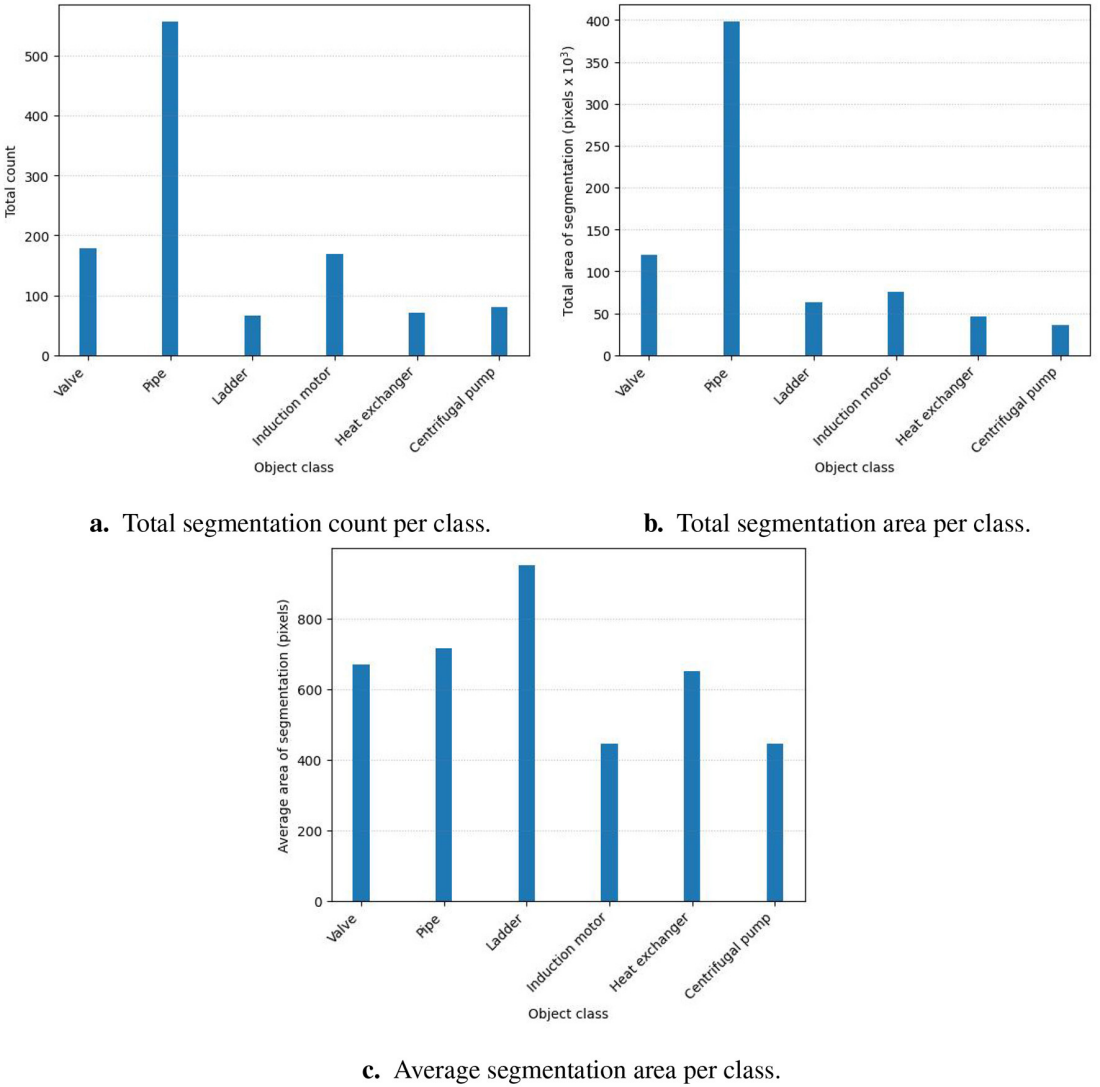
Generated dataset analysis. **(a)** Total segmentation count per class. **(b)** Total segmentation area per class. **(c)** Average segmentation area per class.

Figures 9a–c shows the analysis of the content of the dataset in terms of the total count per class, the total area per class, and finally, the average area per class. The dataset is unbalanced regarding the total segmentation count and total segmentation area. The pipe class has the highest count in the dataset by a margin of 444 against the average of the other classes. The pipe class has the largest total area by a margin of 67226.3 pixels against the average of the other classes. Figure 9c does, however, show a more balanced distribution for the average area of the segmentations per class. The unbalanced dataset was caused by the conclusion drawn from the analysis of the real-world scenes, shown in Figure 5. The analysis showed that pipes are the most common objects in the images.

The deep-learning model chosen to use for instance segmentation is the improved Mask-RCNN with a ResNet-50-FPN backbone (Li et al., 2021) due to its instance segmentation performance in transfer learning approaches. The model was initialized from pre-trained weights using the COCO dataset. The weights for the backbone layers of the model were frozen to leverage the features learned from pre-training on the real-world dataset. A stochastic gradient descent (SGD) optimizer was combined with a decreasing learning rate each epoch as it yields competitive results in transfer learning approaches (Szegedy et al., 2016).

The performance metrics chosen for this study were the IoU and PQ metrics. IoU, as shown in Equation 1, was chosen as it circumvents the class imbalance problem of other metrics. PQ was chosen as it extends IoU to include true positives, false positives, and false negatives in the calculation, as shown in Equation 2. These reasons increase the likelihood of training a high-performing image segmentation model. The higher the IoU and PQ metric values, the better the segmentation performance.

The training was done for five epochs on 300 of the synthetic dataset images (the remaining 29 images were used during evaluation) with a learning rate of 0.004 and a weight decay of 0.0002. The training was done on a computer utilizing an NVIDIA GeForce GTX1650 GPU with 4GB dedicated memory. The average loss value steadily decreased until the final epoch. Additional training did not show a significant decrease in loss; therefore, the number of epochs was not increased beyond five to avoid overfitting, resulting in a final training time of 16 minutes.

Table 2 shows the improvement of performance metrics over each training epoch. Both metrics increased quickly in the first few epochs (as all the training data is new and the learning rate is at its highest). The metrics increased less after the third epoch. The final epoch produces the best result for both metrics.

**Table 2:** Improvement of performance metrics over epochs.

**Metric**	**Epoch 1**	**Epoch 2**	**Epoch 3**	**Epoch 4**	**Epoch 5**
IoU	18.98	31.26	37.04	38.85	39.35
PQ	21.23	39.24	44.33	47.14	49.16

A comparison between the produced segmentation results and existing methods discussed in a recent survey paper (Minaee et al., 2022) is shown in Table 3. The methods in the survey paper were trained on the real-world COCO dataset. The model trained with the synthetic dataset created from our proposed generator reached similar performance metric results.

**Table 3:** IoU results obtained in comparison with recent studies.

**Method**	**Backbone**	**IoU**
DA-Net	ResNet-50	37.9
Our proposed generator	ResNet-50	39.35
EMA-Net	ResNet-50	39.9
AC-Net	ResNet-101	40.1

It would not be accurate to claim that the segmentation model trained with synthetic data outperformed any other model, as results from the survey paper were obtained using a different real-world dataset. However, from this comparison, it can be concluded that training a model using a dataset generated through our proposed method produces similar performance results.

The model using a ResNet-101 backbone obtained the best results among the models from the survey paper, as expected, due to the increased number of layers. However, the increased number of layers does lengthen the training and final inference time and the required computational power.

**Figure 10:**
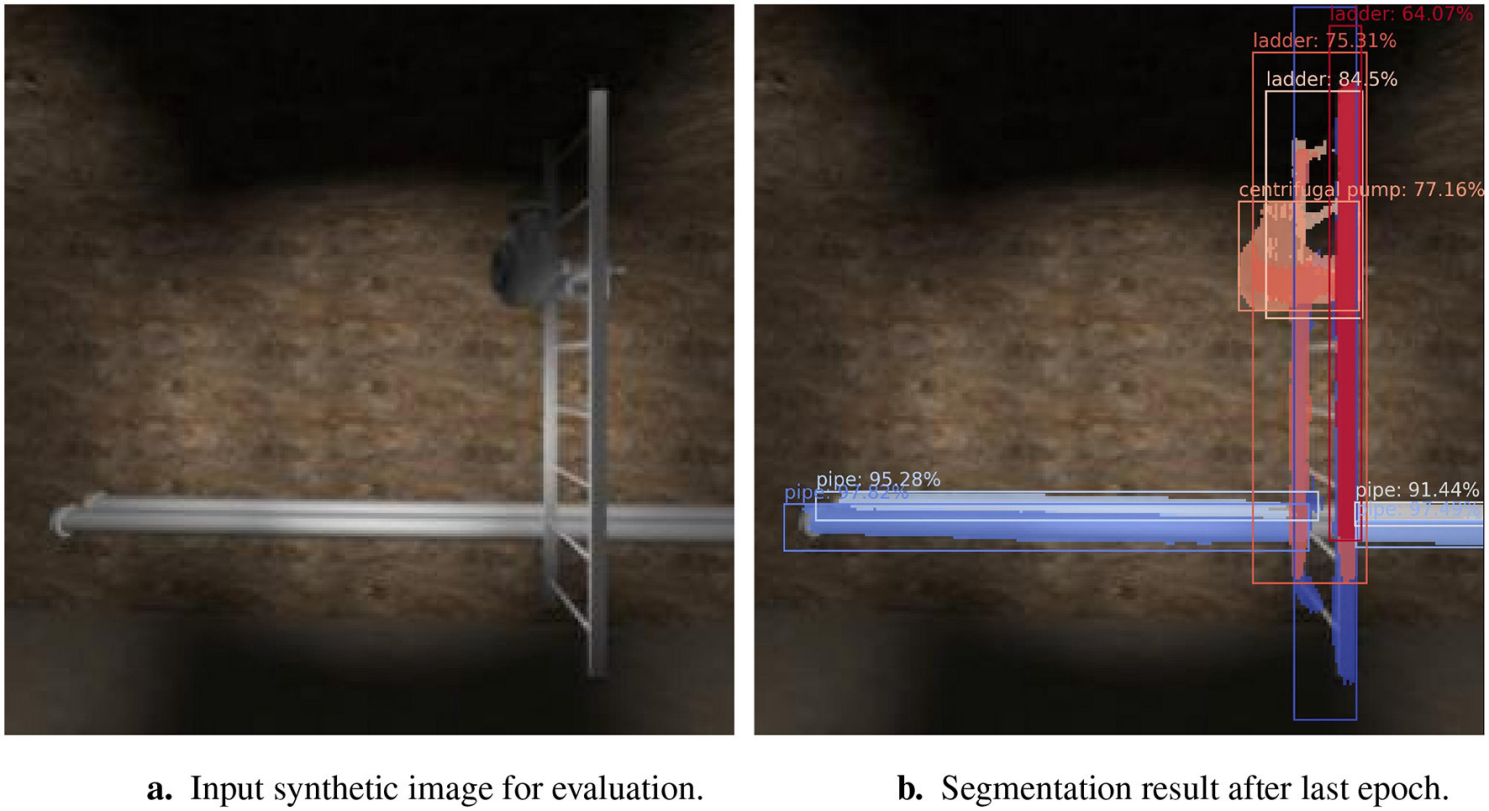
Evaluation result. **(a)** Input synthetic image for evaluation. **(b)** Segmentation result after last epoch.

Figure 10a presents an automatically-generated input image, and Figure 10b shows the segmentation outcome post-final training epoch. All objects in the test image were segmented with confidence scores of at least 64.07%. The two pipes were segmented with high confidence scores (above 91%). However, they were split due to the ladder being in front of the pipes.

Although also located behind the ladder, the centrifugal pump was segmented with a 77.16% confidence score, and the bounding box encompasses almost the entire object. The ladder, although split into multiple segments, was identified with high confidence scores (ranging from 64% to 84%). The performance of the segmentation model in the final round of evaluation was an IoU and PQ score of 39.35 and 49.16, respectively, as shown in Table 2.

**Figure 11:**
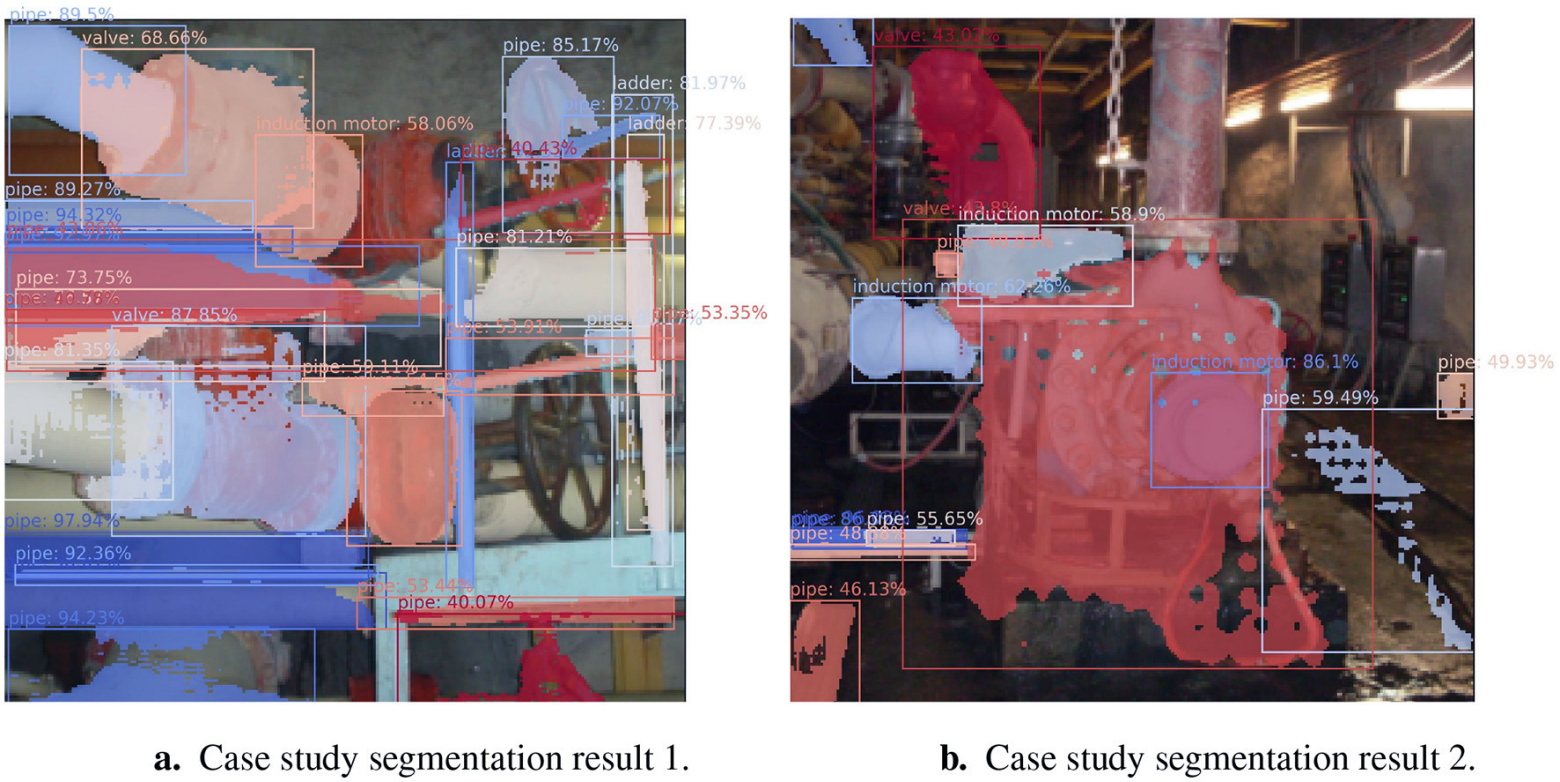
Segmentation results (for the real-world images in Figure 5). **(a)** Case study segmentation result 1. **(b)** Case study segmentation result 2.

Figure 11 shows the segmentation results of the real-world case study images from Figure 5. All segmentations with a confidence score lower than 40% were ignored. In Figure 11a, almost all the pipes were correctly segmented and labeled. The top-left valve was segmented with a confidence score of 68.66% (with a segmentation discrepancy at the top and the right of the valve).

In Figure 11a, the bottom-left valve's profile was mostly segmented with a confidence score of 87.85% (with a discrepancy at the top of the profile). The railing was identified as a ladder, as it resembles the shape of a ladder in the training data. Two valves and three of the pipes were not segmented.

The non-drive end bearing housing in the center of Figure 11b was segmented but incorrectly labeled as an induction motor with a confidence score of 86.1% as it resembles the profile of an induction motor in the synthetic dataset. The profile of the large pump in the middle of the image was segmented well but was mislabeled as it resembles a valve in the training dataset. The valve to the top-left of the image was partially segmented with a score of 43.02%. Various objects (such as the grate to the bottom left and the railway to the bottom right) were mislabeled as they resemble the pipes in the training dataset.

The segmentation performance on the case study images was obtained by manually creating ground truth segmentations and calculating the IoU and PQ metrics for the automatically segmented images. The average IoU and PQ scores achieved on the case study images were 34.15 and 32.41, respectively, as shown in Table 4.

**Table 4:** Final segmentation performance.

**Segmented content**	**IoU**	**PQ**
Evaluation subset of synthetic dataset	39.35	49.16
Case study images	34.15	32.41

The PQ result for the case study images was lower than the IoU result, which is not the case for the synthetic dataset. This is possibly due to the PQ metric being influenced by the false positives, false negatives, and true positives, as some of the objects in the case study images were segmented well in terms of the object profile but were mislabeled as other classes.

The segmentation model's performance on the case study images was slightly lower than that achieved in the final evaluation step during the testing phase with the synthetic dataset. The use of transfer learning to retain the real-world learned features was successful, as no real-world images of the chosen case study were used during model training, and only a slight loss of performance was seen.

All the validation results have shown that the proposed method to generate synthetic data produces datasets usable for training instance segmentation models and that the performance is comparable to those of recent studies using real-world datasets. The validation also showed that the method is cost-efficient, as a relatively low-powered computer was used to train the segmentation model, and the final training time was only 16 minutes.

## 4 Conclusion

The proposed methodology for creating a synthetic dataset of segmented and labeled scenes was applied to a deep-level mining case study. The real-world images contained objects such as pipes, valves, induction motors, and ladders. These images were analyzed, and the synthetic scenes were designed and constructed. 3D models, such as pipes and valves, were utilized to increase the similarity between synthetic and real-world scenes. A camera and raycaster were used to create and label different perspectives within the generated scenes to produce the final synthetic dataset.

The validation comprised utilizing the created dataset in a transfer learning approach on an instance segmentation model while calculating the IoU and PQ metrics after each epoch. The performance metrics increased (initially in large increments) over each epoch and plateaued after five epochs. The inference on the validation dataset showed high confidence instance segmentation and labeling on each object of interest present.

The segmentation performance achieved with the proposed generator was compared to the results reported in a recent survey paper. It showed that the IoU metric results obtained are similar to those reported in the survey paper.

The segmentations of the case study images showed that most of the real-world object's profiles could be segmented and labeled with high confidence and accuracy. Some objects of interest defined for the synthetic dataset were not segmented or mislabeled. However, the results on the real-world data are promising when considering the relatively inexpensive hardware used to train the segmentation model, the final training time, and the fact that a synthetic dataset was used for training.

Future work includes experiments with the proposed generator in various case studies, such as image segmentation for indoor manufacturing or construction sites, to test its generalizability. Investigations into the effects of creating a balanced versus biased dataset for various case studies' real-world scenes can also be conducted to determine a balance between generalizability and segmentation performance. Lastly, testing the dataset generator with various segmentation algorithms is also recommended for future work.

## Data Availability

The raw data supporting the conclusions of this article will be made available by the authors, without undue reservation.
